# Segmental and suprasegmental encoding of speaker confidence in Wuxi dialect vowels

**DOI:** 10.3389/fpsyg.2022.1028106

**Published:** 2022-12-12

**Authors:** Yujie Ji, Yanbing Hu, Xiaoming Jiang

**Affiliations:** Institute of Linguistics, Shanghai International Studies University, Shanghai, China

**Keywords:** Wuxi dialect, confidence, lexical tone, vocal production, formant frequency

## Abstract

**Introduction:**

Wuxi dialect is a variation of Wu dialect spoken in eastern China and is characterized by a rich tonal system. Compared with standard Mandarin speakers, those of Wuxi dialect as their mother tongue can be more efficient in varying vocal cues to encode communicative meanings in speech communication. While literature has demonstrated that speakers encode high vs. low confidence in global prosodic cues at the sentence level, it is unknown how speakers’ intended confidence is encoded at a more local, phonetic level. This study aimed to explore the effects of speakers’ intended confidence on both prosodic and formant features of vowels in two lexical tones (the flat tone and the contour tone) of Wuxi dialect.

**Methods:**

Words of a single vowel were spoken in confident, unconfident, or neutral tone of voice by native Wuxi dialect speakers using a standard elicitation procedure. Linear-mixed effects modeling and parametric bootstrapping testing were performed.

**Results:**

The results showed that (1) the speakers raised both F1 and F2 in the confident level (compared with the neutral-intending expression). Additionally, F1 can distinguish between the confident and unconfident expressions; (2) Compared with the neutral-intending expression, the speakers raised mean f0, had a greater variation of f0 and prolonged pronunciation time in the unconfident level while they raised mean intensity, had a greater variation of intensity and prolonged pronunciation time in the confident level. (3) The speakers modulated mean f0 and mean intensity to a larger extent on the flat tone than the contour tone to differentiate between levels of confidence in the voice, while they modulated f0 and intensity range more only on the contour tone.

**Discussion:**

These findings shed new light on the mechanisms of segmental and suprasegmental encoding of speaker confidence and lack of confidence at the vowel level, highlighting the interplay of lexical tone and vocal expression in speech communication.

## Introduction

Imagine a situation where a student on a language-learning class asks the lecturer what a specific written word is pronounced because that word is printed in a visually-unrecognized manner. When responding to students, the lecturer may find themselves not sure what the word is. This is when the lecturer replies with his or her own pronunciation of the word to convey their knowledge toward how they evaluate that specific situation.

In daily interactions, speakers often assess whether the event they perceive is true and whether what they say is correct, and they show evidence on their evaluation of things in their statements. Speakers may use the epistemic modality to convey their feeling of (un)knowing about what is proposed (Swerts and Krahmer, 2005). Except for the modal auxiliaries and modal adverbs (Coates, 2012), epistemic modality encompasses a wide range of linguistic forms that feature a specific pattern of prosodic and paralinguistic cues, which are valuable resources for speakers to use to indicate the speaker’s confidence or lack of confidence in the truth of the proposition expressed in the discourse. In face-to-face communication, human have an intuition about how confident our conversational partner is about what they are saying.

Vocal confidence expressions serve as “evidentiality” devices for inferring the reliability, correctness, or truth value of what is expressed from a speaker’s tone of voice (Caffi and Janney, 1994; Jiang and Pell, 2015). In particular, a speaker’s possession of confidence is typically encoded by external cues that provide evidence for the speaker’s knowledge about the self-evaluated correctness or truth value of his own statements (London et al., 1970a,b, 1971; Scherer et al., 1973). In contrast, the speaker’s lack of confidence or doubt (with only 50% certainty about whether what is said is true) indicates a person’s negative attitude or hesitation about a fact or opinion, which is marked by cues that supply signs of untrustworthiness (the lack of moral value of showing remorse or taking responsibility for having done something wrong) or lack of credibility (the perceived believability of information that leads to the listener’s feeling of trust; Kuhlen et al., 2015; Belin et al., 2017; Jiang and Pell, 2017).

Previous acoustic-phonetic studies have been conducted from different perspectives regarding whether confidence is defined according to the speaker intention or the listener perception. In the first group of study, speakers were instructed experienced to utter sentences in a confident vs. unconfident way, after which acoustic analysis was performed by measuring different prosodic characteristics of the speaker’s voice based on which level of confidence the speakers’ intended. The results showed that speakers often spoke with a higher pitch and at a greater intensity when they intended to be confident (Scherer et al., 1973; Van Zant and Berger, 2020). In a second set of works, the same group of vocal stimuli was judged on speaker confidence by an independent group of listeners, and the acoustic analysis was performed based on the regrouping of the stimuli according to the listener’s perception. Results showed a distinct pattern of pitch, intensity, and temporal features according to the perceived levels of confidence: the confident expressions were highest in the variation of fundamental frequency (f0), mean amplitude, and amplitude range, but were lower than the unconfident ones in the mean f0, emphasizing the set of acoustic features that listener showed the sensitivities to Jiang and Pell (2014, 2017). In addition, a smaller set of studies directly manipulated the acoustic parameters of the speech and assessed the listener’s perceived confidence. These studies showed that the lower pitch can elicit perceptions of higher confidence (Guyer J. 2016).

Differential approaches to determining acoustic-phonetic features based on speaker intended expression or listener perception is how speech materials are selected for acoustic analysis. In the former approach of analysis, the study utilized listeners’ perception to validate that the differences in the acoustic features are indeed attributed to the speaker intention. According to the latter approach, the material was regrouped based on perception results, and the regrouped stimuli could only reflect what listeners’ commitment but not speakers’ own intention. Additionally, while in ideal cases, the speaker and the listener are convergent in the use of communicative cues, in many cases, such convergence is not reached and the encoding and the decoding processes seem to rely on a partially-independent set of cues (Jiang and Pell, 2016, 2017, 2018). In Brunswik’s lens model (Brunswik, 1956), acoustic cues in the voice are understood by listeners as probabilistic and partly redundant. The accurate perception of speaker confidence usually depends on both verbal and vocal cues, which can be weighed differently by listeners (Jiang and Pell, 2016). Crucially, listeners are thought to rely on these cues in a partly interchangeable manner (Juslin and Laukka, 2003).

While epistemic and social meanings have been demonstrated to be encoded at the suprasegmental level of speech, they are often found to occur at the segmental level in a much smaller spoken unit. In a study by Laukka et al. (2005) on vocal emotion, it was noted that the first formant (F1) of the stable portion of vowels can predict the level of affective activation. Another study revealed that the first and second formant (F2) of the vowels was influenced by different affect dimensions (Goudbeek et al., 2009). For example, monophthongs of higher-level of arousal resulted in a higher mean F1 than those of lower-level of arousal, whereas monophthongs of positive valence resulted in higher mean values of F2 than those of negative valence. It was also found that adults who stuttered had significantly greater F2 frequency fluctuations when speaking in situations that elicited increases in arousal and unpleasantness. They also showed that those who did not stutter showed little change in F2 fluctuations across varied emotion categories (Bauerly, 2018). Despite that the emotional and epistemic meaning of speech could differ in many aspects, someone argues that the expression of emotions enables speakers to communicate powerful messages to others, which in turn may have a consequence on their attitudes and perceived stances (Guyer J. J. 2016). Delivering an emotional message in persuasive vs. neutral manner altered the voice onset time of consonants (Banzina, 2021; Jiang and Lu, 2021). The complex interplay between the emotions expressed in the voice and the speaker confidence toward the emotional messages suggests that the alterations in the formant frequencies may also be shown in the confidence-related speech. Thus, the present study aimed to examine whether the levels of speaker confidence can be encoded at the segmental level.

More interestingly, the acoustic realization of lexical tones and vocal expressions of social information could involve similar mechanisms. Not only the intonation that conveys social information is realized by acoustic parameters such as the level and variation of f0, but also is the lexical tone in tonal language reflected in the nature of f0 (Eady, 1982; Cutler and Chen, 1997). In tonal languages, the lexical tone is treated as pitch patterns as a contrastive feature. One intriguing aspect of tonal language/dialect is that the intonation system is independent from the lexical tone, although both elements can be expressed by the f0 contour to symbolize the change. This means that some acoustic features such as the f0 contour carries the identifying functions of both linguistic and paralinguistic information. To understand the encoding mechanism of vocal expressions in speech communication, it is essential to investigate the interplay between the lexical tone and intonation of certain emotional or pragmatic function in the context of tonal language/dialect.

Despite that many studies examined the acoustic realizations of different lexical tones (e.g., pitch contour, and duration), there were rare investigations on how the lexical tone could modulate the way social information is encoded in the expressive tone at the segmental level. Chao (1933) proposed two items to distinguish two interplay types of tone and intonation addition patterns: simultaneous addition and successive addition. The simultaneous addition refers to the tones that are the algebraic sums of two factors: the original lexical tone and the sentence intonation proper. The successive addition refers that a rising or falling intonation of a clause is not added simultaneously to certain syllables but added on successively after the lexical tones are completed. The function of the successive addition boundary tone is to express the speaker’s emotion rather than to convey linguistic contents. An empirical study investigated how the lexical tone and affective tone interacted in Mandarin Chinese, using monosyllabic emotional utterances as materials (Li et al., 2011). It was found that the tonal space (with all f0 values mapped into a five-point scale), the edge tone (the pattern among the tone and the intonation being added up in emotional speech), and the length of monosyllabic materials differed greatly between seven emotions. In other words, the f0 pattern of lexical tones was affected by the emotional intonation. Furthermore, researchers pointed out that boundary tones of emotional intonation are more appropriately characterized by both traditional boundary tone features and successive addition tone features (Li et al., 2012). For instance, the “disgusting” sound had a “falling” addition tone following the lexical tone of the last syllable, assembled as successive addition tones. These analyses or findings have strongly suggested that the lexical tone and the expressive tone co-constrain the acoustic encoding of social information in speech at the suprasegmental level (e.g., f0 features).

Similar to Mandarin Chinese, Wuxi Dialect, as a member of Wu dialects, has a rich segmental system that consists of 27 consonants, 44 vowels, and eight tones. For instance, Wuxi vowels contain 19 monophthongs, 21 diphthongs, and four triphthongs (Wen, 1996; Cao, 2003). Considering that the Wuxi dialect also has a rich system of tones, it is likely that the vocal expression of confidence in this dialect could also show a pattern of successive or simultaneous addition to the lexical tones. In particular, the tonal context (a flat tone or a contour tone) could modulate the acoustic encoding of vocally-expressed confidence in the Wuxi dialect.

Some studies reported acoustic encoding of vocal expressions from a limited number of speech materials spoken by a larger number of speakers (Pell et al., 2009; McAleer et al., 2014; Ponsot et al., 2018), which had the advantage of considering inter-speaker variability to reveal a generalizable pattern across speakers. However, it could also suffer from poor generalizability across items concerning limited materials. Other reports focused on a larger number of materials spoken by a smaller number of speakers (typically 4–8 speakers, e.g., Pell et al., 2009; Liu and Pell, 2012; Hellbernd and Sammler, 2016; Jiang et al., 2017, 2020; Caballero et al., 2018). The method using numerous materials from limited speakers has the advantage of better generalizability across spoken materials and the disadvantage of lack of inter-speaker variability.

In the present study, 20 different vowels were included for the analysis, which aimed to increase the generalizability across the vowel acoustic space. Additionally, four speakers (two males and two females, from the middle-aged to the elderly) were chosen for this study. To compensate for the relatively lower generalizability across speakers, the speakers were selected to increase the speaker variations in social identities (such as biological sexes and ages) as much as possible.

Considering that related previous studies were mainly focused on the suprasegmental level of vocal expression in sentences in non-tonal languages such as English, the present study aimed to investigate how tonal-language (i.e., Wuxi dialect) speakers encode social intentions in their voices at both segmental and suprasegmental levels. To achieve our purpose, therefore, we generated a corpus with four native Wuxi dialect speakers (i.e., two females and two males) expressing different levels of confidence (three levels: confident, unconfident, and neutral-intending) in different lexical tones (two levels: flat vs. contour tone) through vowels. We measured which acoustic cues individuals use at the segmental level (and also at the suprasegmental level) to encode confidence levels in their voices and tested the way in which these acoustic-phonetic features of confidence were influenced by lexical tones.

The acoustic features the present study focused are as followed. The segmental features included the first two formants (F1 and F2; Laukka et al., 2005; Goudbeek et al., 2009; Ji and Jiang, 2021; Salais et al., 2022); the suprasegmental features included (1) The fundamental frequency (f0); (2) The sound intensity (dB); and (3) Duration (Scherer et al., 1973; Jiang and Pell, 2014, 2017; Guyer J. 2016; Van Zant and Berger, 2020). The present study focused on these acoustic parameters because the social information intended in the speaker voice has been associated with these segmental and suprasegmental features in related studies.

Previous studies showed the association between the increased arousal and higher mean F1 and the association between the positive potency and higher mean F2 in the speech. It is expected that confident and unconfident voices could lead to higher F1 compared to neutral voices, and confident voices could be associated with higher F2 than neutral and unconfident voices. Given that prosodic features such as pitch and intensity have been shown to reliably differentiate different speaker confidence, it is expected that the confident voice would show lower fundamental frequency and greater intensity as compared with the unconfident voice, as such finding can be extended from English at the sentence level to a type of eastern Chinese-dialect at the level of a smaller segmental unit. Considering that lexical tone plays a role in the expression of emotions in previous studies, an interaction between speakers’ intended confidence and lexical tone is expected to occur in the size of f0, which means that the lexical tone affects the acoustic representation of vocal expression of speaker confidence.

## Materials and methods

### Participants

Four native Wuxi-dialect speakers were invited to produce sentences in different levels of confidence in their native dialect. Only middle-aged and elderly adults but not young adults were selected as speakers because studies have shown that the Chinese dialect pronunciation remained more stable in the middle-aged and elderly populations (Liu and Chen, 2018; Zhang, 2020). Moreover, speakers of certain age ranges were selected to increase the generalizability of the findings by increasing the speaker variations in social identities (such as biological sexes and ages). All speakers (Mean Age = 64.25 years, SD = 17.10 years, and two females) were all born and raised up in Wuxi, a city located in the Jiangsu Province in China, where local residents speak Wuxi Dialect as their native tongue (Cao et al., 2003). All speak Mandarin but did not pick it up until 5 years old. All reported to speak only Wuxi dialect at home and use dialect to communicate more often than Mandarin during work. None of the speakers had lived outside of Wuxi consecutively for over 2 years. The mean self-reported proficiency of the four speakers was 6.25 (SD = 0.5) for speaking and 6.75 (SD = 0.5) for listening Wuxi Dialect, and was 5.25 (SD = 1.5) for speaking and 7 (SD = 0) for listening Mandarin Chinese (out of seven-point scale, with 1 the least proficient and 7 the most proficient). All reported to receive formal education for 12 years. All speakers reported to have normal hearing and none had suffered any previous neurological or speech disorders. Speakers were not selected for having previous training or experiences in professional acting or public speaking. This study was approved by the Ethics Committee of the Institute of Linguistics from the Shanghai International Studies University.

### Materials

**Figure 1:**
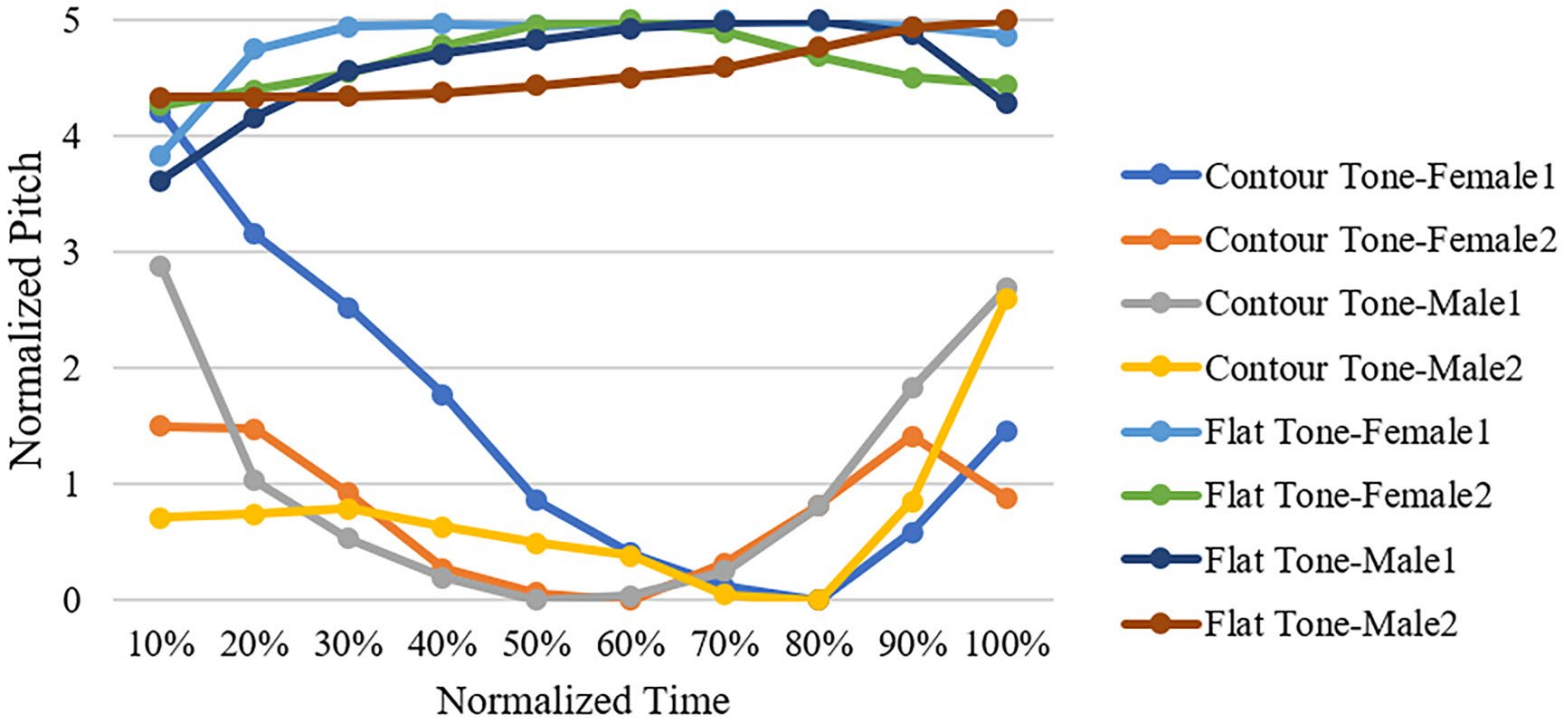
Four speakers neutral-intending expression of vowel /a/ with contour tone and flat tone normalized in a five-point scale.

To eliminate the potential effect of local consonants on the subsequent vowels, word materials for production were selected with zero-consonant. Vowels were selected exhaustively based on the phonological system of the Wuxi dialect (Cao, 2003) to enrich the types of vowel materials and increase the degree of vowel variation. The selected totally 20 vowels consisted in 10 monophthongs (i, u, y, ɚ, a, ʌ, ʊ, ɛ, ã, ɒ̃) and 10 diphthongs (ia, ua, iʌ, yʊ, uɛ, ei, əɯ, iã, uã, uɒ̃). Despite covering such a variety of vowels, the present study was interested in the overall patterns of different vowels at different levels of confidence instead of the differences in the vowels themselves. Two lexical tones (i.e., the flat tone and the contour tone) were selected as target tones (see Figure 1). These two tones were chosen for two reasons: First, all vowels can be produced in both flat tone and contour tone contexts, to ensure vowels in target tones correspond to real words in Wuxi dialect to the maximal extent^1^; Second, these two tones are representative in terms of the fundamental frequency patterns, with the flat tone having a stable fundamental frequency throughout the vowel, and the contour tone having a constantly changing fundamental frequency throughout the vowel. A previous study demonstrated that formant peaks contributed to the high level tone and the third tone in Mandarin Chinese (Zhang et al., 2021). In Wuxi dialect, the flat tone is similar to the high-level tone in Mandarin while the contour tone is similar to the third tone in Mandarin which starts at the low tone with a slight fall and then rises to a high pitch.

Altogether, 40 different words were selected to form the production list for the elicitation (Supplementary Table S1, see Supplementary materials). Carrier sentences were created such that each word was embedded in a sentence “This word is ‘X’,” and to ensure the zero-consonant vowels were preceded by a local linguistic context which was semantically neutral. Therefore, in total there were 480 stimuli (4 speakers × 3 confidence levels × 20 vowels × 2 lexical tones).

### Recording and elicitation procedure

Speakers were seated in a quiet room in front of the TroyStudio portable sound absorption equipment which aimed at reducing sound reverberation and environmental noise. The vocal stimuli were recorded by a TASCAM-DR-07X recorder (with a sampling rate of 44.1 kHz, 16 bit, mono, input level of −9 dBV). The distance between mouth and the microphone was approximately 15 cm and was ensured for each speaker. To facilitate the production of the vocally-expressed confidence, speakers were instructed to produce each sentence twice with a certain level of confidence by responding to the same question from a native Wuxi dialect female confederator in a mini-dialog format (e.g., Question: What is the word? Answer: This word is “X”; Jiang and Pell, 2014, 2017, 2018). The target vowel was the new information in the answer which corresponded to the wh-constituent in the questions, which aimed at inducing natural vowels for subsequent acoustic analysis (Waters et al., 2021). The question was asked in a neutral tone of voice. The answer was produced in a certain level of confidence. The speakers were instructed to articulate the word clearly and to communicate the target level of confidence directly to the confederator and to avoid simply reading out the sentences.

The vocal stimuli were recorded in separate blocks, in each of which a certain intended level of confidence was elicited. Such procedure has proven successful to elicit a stable level of speaker expression across sentences. In the confident condition, the speakers were instructed to produce the sentence with 100% certainty that the word they said in the sentence was true. In the unconfident condition, the speakers produced the sentence with the knowledge that only in 50% cases the word they said was true. The unconfident expression was not elicited through questions given that the encoding of linguistic question was not the same as the vocal expression of lack of speaker confidence. For instance, the speaker could simply lengthen the production of certain constituents to mark their lack of confidence (Jiang and Pell, 2017). To elicit a condition which lacked in any level of explicitly-encoded speaker confidence, speakers were also instructed to produce a corresponding set of neutrally-intending sentences. In this condition, the speakers were encouraged to produce utterances “without feeling any particular emotion or attitude” toward the content of the sentence. At no time did the confederator provide an explicit model of how intended target meanings should be expressed. For confident and unconfident blocks, the speaker was additionally instructed to convey the intended level of confidence throughout the sentence. The order of the three recording blocks (confident, unconfident, and neutral) were randomized across speakers with the exception that the block for the neutrally-intending expressions always preceded the blocks of confident and unconfident expressions. Breaks were inserted between blocks to ensure a successful transition between modes of different levels of confidence. The repetition of each sentence was initially evaluated by a native Wuxi-dialect speaker to select the best exemplar per item/speaker, based on her intuition to decide which item better conveyed the intended target level of confidence, and to discard the items that sounded unnatural and/or had speech errors.

To ensure that the three levels of speaker’s intended confidence were perceived as different, 16 participants who did not participate in the production task listened to each vowel and rated the speaker’s level of confidence on a seven-point scale (1 = not at all confident; 7 = very much) for all stimuli. The mean rating was 3.93 (SD = 1.62) for the unconfident expression, 4.20 (SD = 1.46) for the neutral expression and 4.57 (SD = 1.51) for confident ones. One-way ANOVA showed that the three levels of speaker’s intended confidence was perceptually different [*F* (2,7,526) = 118.42, *p* < 0.001; Bonferroni post-test, *t*s > 6.43, *p*s < 0.001].

### Data analysis

Based on the preliminary screening, a total of 477 recordings including both monophthongs and diphthongs were subjected to further analysis, with one diphthong of the flat tone produced in the confident expression of one female speaker and one monophthong of two lexical tones produced in the unconfident expression of the other female speaker were discarded due to pronunciation errors.

Both the segmental features that distinguish vowel units and the suprasegmental features that are superimposed on these units were analyzed on target vowels in order to show different levels of acoustic features of vowels expressed in different intended levels of confidence.

#### Segmental features

To quantify F1 and F2, we labeled the stable articulation of the vowels based on the selected stimuli in TextGrid with Praat (Version 6.1.52) before extracting the mean values of F1 and F2. For the monophthongs, the stable articulations were labeled; whereas for the diphthongs, the stable articulations of the first and the second vowels were separately labeled. The Praat script^2^ was adapted to extract mean formants (Hz) of the stable section of the particular vowels labeled in the Textgrid Tier for both monophthongs and diphthongs.

#### Prosodic features

The prosodic features included: the mean fundamental frequency (mean f0, in Hz), the range of fundamental frequency (f0 variance, in Hz), and the mean sound intensity (mean intensity, in dB), the range of sound intensity (intensity variance, in dB) for both monophthongs and diphthongs, duration (in ms) for monophthongs only^3^. The same stable parts for the vowels as in the analysis of segmental features were used to obtain prosodic features except duration. The entire vowel articulation was labeled to define the duration for monophthongs. Formant and prosodic values extracted from the first and the second vowels of the same diphthong were treated as two separate parts. The *ProsodyPro* tool (Xu and Prom-On, 2014) was used to extract duration, intensity (mean intensity, maximum intensity, and minimum intensity) and fundamental frequency (mean f0, maximum f0, and minimum f0) of the vowel stimuli. The intensity range and the f0 range were then calculated by subtracting the minimum value from the maximum value.

A normalization procedure was applied to all prosodic features of each stimuli before comparing between speakers (Pell et al., 2009; Liu and Pell, 2012; Jiang and Pell, 2017). The mean fundamental frequency of each speaker’s articulation naturally differs, and the absolute differences in f0 range vary as an index of the speaker’s meanf0. There is evidence that when speaking in a non-emotional manner, each speaker has to a highly stable “resting frequency” or end-point f0 at the end of their utterances which is characteristic for that individual (Menn and Boyce, 1982; Pell et al., 2009). In order to correct for the individual difference in a speaker’s mean voice pitch, all f0 measures (mean, maximum, and minimum f0) were normalized in relation to the individual “resting frequency” of each speaker (i.e., the average minimum f0 value of all neutral stimuli produced by that speaker). Measures of normalized f0 range were then calculated by subtracting the normalized minimum f0 values from the normalized maximum f0 values. The same method was applied to the normalization of the intensity values of each speaker. The normalized duration for monophthong or diphthong was obtained in relation to the individual “resting production length” of each speaker (i.e., the average mean duration of all neutral stimuli for monophthongs or diphthongs produced by that speaker).

#### Statistical analysis

Statistical modelings were conducted for segmental and prosodic features separately. Considering the correlations among our dependent variables (Jiang and Pell, 2014, 2017), multiple ANOVA (MANOVA) were used to reduce the joint error rate and to achieve greater statistical power compared to a series of ANOVA tests (Matuschek et al., 2017; see also https://statisticsbyjim.com/anova/multivariate-anova-manova-benefits-use/). To ascertain whether speaker confidence differed in the linear composition of acoustic features, MANOVAs were conducted on the linear composition of formant features and of suprasegmental features (f0 and intensity values) separately.

To determine the effects of Lexical Tone, Speaker Confidence and their interaction(s) on each independent acoustic feature, linear mixed effects models (LMMs) were separately conducted on each segmental and suprasegmental feature. The model selection procedure started with a baseline model including only by-subject and by-vowel item random intercepts. Predictors were then added in a step-wise fashion to determine the model fit. Model comparisons were conducted using chi-squared tests of model log-likelihoods. The predictor was dropped from the model when it did not yield significant improvement in the model comparison (Ip and Cutler, 2020). The AICs (Akaike Information Criterion) of added models were compared. Compared with the baseline model, the best fitting model contained significant effect of Lexical Tone, Speakers Confidence and their interaction for model of F1 [*χ^2^*(2) = 7.42, *p* = 0.025], F2 [*χ^2^*(2) = 11.11, *p* = 0.049], mean f0 [*χ^2^*(2) = 19.00, *p* < 0.001], range of f0 [*χ^2^*(2) = 17.04, *p* < 0.001], mean intensity [*χ^2^*(2) = 11.96, *p* < 0.001], and range of intensity [*χ*^2^(2) = 8.20, *p* = 0.012]. The fixed factors were Lexical Tone and Speakers Confidence. The random factors were Subjects and Vowel Items.

y^4^ ~ lexical tone*levels of confidence + (1|Subject) + (1|Item)

All data were analyzed using linear mixed effects models (LMMs) within the *lmerTest* packages of R (Version 3.1.3, https://github.com/runehaubo/lmerTestR). Considering the sample size per speaker confidence per lexical tone was 120^5^ for all models except for the model of duration (*n* = 80^6^), the *p*-values for fixed effects were tested by parametric bootstrapping^7^ using function *mixed()* from R package “afex” (nsim = 10,000; Singmann, 2019).

Considering the complexity of acoustic parameters in the LMMs, the current study put the results of statistics results into tables to ensure the conciseness and intuitiveness of the results.

## Results

### Segmental features

Table 1 demonstrated the mean F1 and F2 values computed for all vowels across lexical tones and levels of speaker confidence. The MANOVA on the linear combination of the two formant parameters showed a significant effect of Speaker Confidence [Pillai’s Trace =0.03, *F* (2,702) =5.30, *p* < 0.001, *η^2^_p_* = 0.01]. The models for the effect of Lexical Tone did not reach significance [Pillai’s Trace = 0.001, *F* (1,708) =0.31, *p* = 0.735, *η^2^_p_* = 0.0008].

**Table 1:** Mean and SD of mean F1 and F2 values (in Hz) in different lexical tones averaged between speakers.

	F1		F2	
	Flat tone	Contour tone	Flat tone	Contour tone
Confident^a^	687.22 (291.85)	629.78 (305.07)	1667.49 (562.71)	1720.40 (589.64)
Unconfident	543.66 (273.19)	572.55 (295.99)	1613.19 (581.39)	1635.03 (611.89)
Neutral	567.27 (273.36)	574.89 (283.19)	1565.96 (614.03)	1593.67 (610.40)

Standard deviations were shown in brackets. ^a^Sample size per cell was 120, except that for vowels of a flat tone, the sample size was 118 for the confident expression and was 119 for the unconfident expression; for those of a contour tone, the sample size was 119 for the unconfident expression, given that non-standard incorrect pronunciations were discarded.

**Figure 2:**
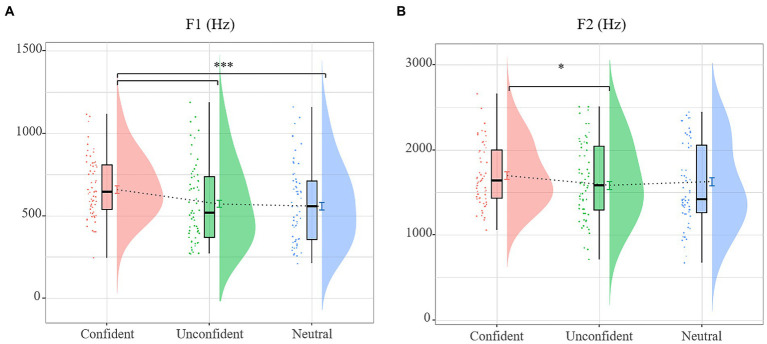
Raincloud plots for formant features showing the main effect of speaker confidence. **(A)** F1 and **(B)** F2 values per confidence level for all vowels.

To ascertain the potential effect of Speaker Confidence and its interaction with Lexical Tone, the LMMs were separately built on mean values of F1 and F2 (see Table 2). The F1 model revealed a significant main effect of Speaker Confidence, suggesting that the confident expression revealed a larger F1 than the unconfident and the neutral-intending expression, and the unconfident did not differ from neutral-intending expression (see Figure 2A).

**Table 2:** LME model performances for formant features.

Formant features	Effect	Chisq	P-value	Contrast	Estimate	SE^b^	*t*	P-value^a^	95%CI
F1	Lexical Tone	1.57	0.207	Contour—Flat					
	Speaker Confidence	27.89	***	Conf—Neut	87.5	20.4	4.29	***	[38.6,137.0]
				Conf—Unconf	99.5	20.5	4.87	***	[50.4,149.0]
				Neut—Unconf	12.0	20.4	0.59	1.00	[−37.0,61.0]
	Lexical Tone × Speaker Confidence	4.91	0.092						
								
F2	Lexical Tone	0.02	0.890						
	Speaker Confidence	7.63	0.026**	Conf—Neut	116.7	42.5	2.75	0.019	[14.7,218.6]
				Conf—Unconf	68.5	42.6	1.61	0.324	[−33.7,170.7]
				Neut—Unconf	−48.1	42.5	−1.13	0.773	[−150.1,53.8]
	Lexical Tone × Speaker Confidence	0.10	0.955						

^a^Significance levels under Bonferroni-corrections: *p < 0.05; **p < 0.01; ***p < 0.001. ^b^SE: standard error.

The F2 model revealed a significant main effect of Speaker Confidence, suggesting that the confident expression revealed a larger F2 can only be seen between confident vs. neutral-intending expression (see Figure 2B).

In summary, the speakers raised both F1 and F2 in the confident level (compared with the neutral-intending expression). Additionally, F1 can distinguish between the confident and unconfident expressions.

### Prosodic features

We examined whether speakers utilized prosodic cues to express levels of confidence under two different lexical tones in the same two steps: MANOVAs and LMERs. In Table 3, the means and SDs for the prosodic values of vowels by all factor levels (lexical tones and levels of speaker confidence) are presented.

**Table 3:** Means and standard deviations of the normalized pitch, intensity, and duration measures in different lexical tones averaged across speakers.

	Mean F0		F0 range		Mean intensity		Intensity range		Duration^b^	
	Flat tone	Contour tone	Flat tone	Contour tone	Flat tone	Contour tone	Flat tone	Contour tone	Flat tone	Contour tone
Confident^a^	0.46 (0.20)	0.003 (0.22)	0.13 (0.13)	0.29 (0.23)	0.10 (0.07)	0.05 (0.06)	0.08 (0.05)	0.09 (0.07)	0.88 (0.18)	1.17 (0.18)
Unconfident	0.59 (0.23)	0.02 (0.22)	0.13 (0.15)	0.30 (0.21)	0.05 (0.08)	0.02 (0.06)	0.08 (0.06)	0.07 (0.05)	0.94 (0.19)	0.94 (0.19)
Neutral	0.29 (0.16)	−014 (0.13)	0.12 (0.12)	0.18 (0.13)	0.08 (0.06)	0.02 (0.05)	0.08 (0.05)	0.07 (0.05)	0.84 (0.16)	0.84 (0.16)

Standard deviations were shown in brackets. ^a^Sample size of f0 and intensity features was 120 (40 items for monophthongs and 80 items for diphthongs), except that: for vowels of a flat tone, the sample size was 118 for the confident expression, 119 for the unconfident and neutral-intending expression; for those of a contour tone, the sample size was 117 for the confident expression, 118 for the unconfident expression, and 119 for the neutral-intending expression given non-standard incorrect pronunciations. ^b^Sample size of duration was 80, except that for monophthongs of a contour tone, the sample size was 78 for the unconfident expression; for those of a flat tone, the sample size was 79 for the unconfident expression.

The MANOVA was first built for the effect of Speaker Confidence on the linear combination of four prosodic parameters, including mean f0, f0 range, mean intensity, and intensity range. The model showed a significant effect of Speaker Confidence [Pillai’s Trace =0.26, *F* (2,707) = 25.96, *p* < 0.001, *η^2^_p_* = 0.12]. The MANOVA also showed a significant effect of Lexical Tone [Pillai’s Trace = 0.61, *F* (1,708) =277.13, *p* < 0.001, *η^2^_p_* = 0.61]. Both Speaker Confidence and Lexical Tone significantly modulated the linear combination of the prosodic parameters.

**Figure 3:**
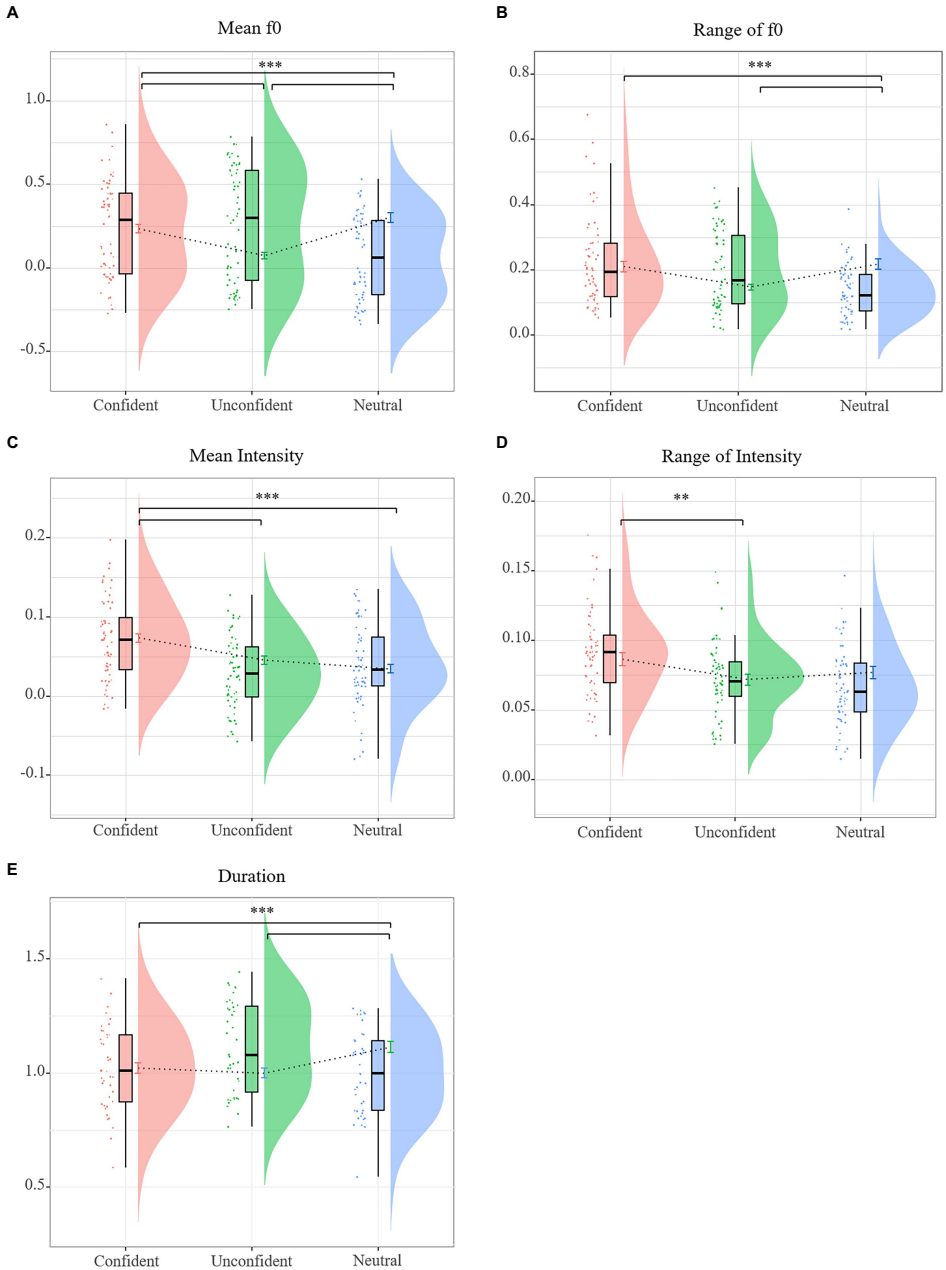
Raincloud plots for prosodic features showing the main effect of speaker confidence. **(A)** mean f0, **(B)** f0 range, **(C)** mean intensity, **(D)** intensity range per confidence level for all vowels, and **(E)** duration for monophthongs.

**Figure 4:**
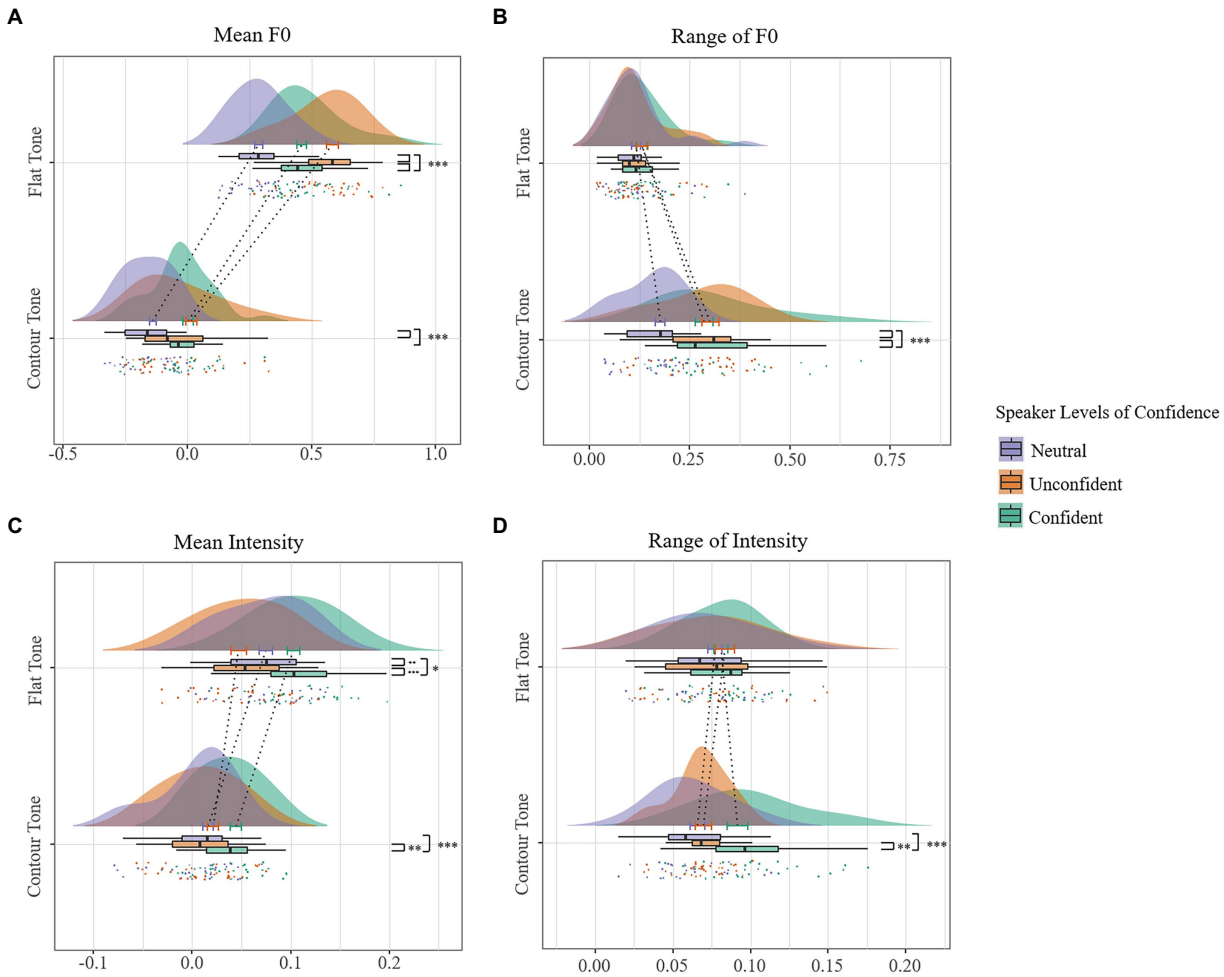
Raincloud plots for prosodic features showing the interaction of speaker confidence and lexical tone. **(A)** mean f0, **(B)** f0 range, **(C)** mean intensity, and **(D)** intensity range per lexical tone per confidence level for all vowels.

To show the potential effect of Speaker Confidence and its interaction with Lexical Tone, the LMMs were separately built on each prosodic factor (see Table 4). The mean f0 model revealed a significant main effect of Speaker Confidence (see Figure 3A), suggesting that the mean f0 was largest in the unconfident expression, seconded by the confident, and was smallest in the neutral-intending expression. The model revealed a significant main effect of Lexical Tone, suggesting that the mean f0 was significantly larger in vowels of a flat tone than those of a contour tone. The Speaker Confidence x Lexical Tone interaction was significant (see Figure 4A). For vowels of a flat tone, the mean f0 differed among three levels of confidence, with the mean f0 largest in the unconfident expression, followed by the confident, and smallest by the neutral-intending expression; for those of a contour tone, the mean f0 was larger in the unconfident than in both the confident and the neutral expression and the confident did not differ from neutral-intending expression.

**Table 4:** LME model performances for normalized prosodic features.

Prosodic features	Effect	Chisq	*p* value^a^	Contrast		Estimate	SE^b^	*t*	*p* value	95%CI
Mean F0	Lexical Tone	700.06	***	Contour—Flat		−0.49	0.01	−34.91	***	[−0.51, −0.49]
	Speaker Confidence	166.69	***	Conf—Neut		0.16	0.02	9.28	***	[0.12,0.20]
				Conf—Unconf		−0.07	0.02	−4.04	***	[−0.11,-0.03]
				Neut—Unconf		−0.23	0.02	−13.36	***	[−0.27,-0.19]
	Lexical Tone × Speaker Confidence	19.02	***	Contour tone	Conf—Neut	0.14	0.02	5.89	***	[0.08,0.20]
					Conf—Unconf	−0.01	0.02	−0.58	1.00	[−0.07,0.04]
					Neut—Unconf	−0.16	0.02	−6.49	***	[−0.21,-0.10]
				Flat tone	Conf—Neut	0.17	0.02	7.24	***	[0.12,0.23]
					Conf—Unconf	−0.12	0.02	−5.14	***	[−0.18,-0.07]
					Neut—Unconf	−0.30	0.02	−12.41	***	[−0.36,-0.24]
F0 range	Lexical Tone	107.75	***	Contour—Flat		0.13	0.01	10.86	***	[0.11,0.15]
	Speaker Confidence	27.21	***	Conf—Neut		0.06	0.01	4.30	***	[0.03,0.10]
				Conf—Unconf		−0.01	0.01	−0.52	1.00	[−0.04,0.03]
				Neut—Unconf		−0.07	0.01	−4.82	***	[−0.10,-0.04]
	Lexical Tone × Speaker Confidence	16.61	***	Contour tone	Conf—Neut	1.10e-01	0.02	5.37	***	[0.06,0.16]
					Conf—Unconf	−1.50e-02	0.02	−0.73	1.00	[−0.06,0.03]
					Neut—Unconf	−1.25e-01	0.02	−6.09	***	[−0.17,-0.08]
				Flat tone	Conf—Neut	1.48e-02	0.02	0.72	1.00	[−0.03,0.06]
					Conf—Unconf	−5.38e-06	0.02	0.00	1.00	[−0.05,0.05]
					Neut—Unconf	−1.48e-02	0.02	−0.73	1.00	[−0.06,0.34]
Mean intensity	Lexical Tone	104.67	***	Contour – Flat		−0.05	0.00	−10.70	***	[−0.06, −0.04]
	Speaker Confidence	54.24	***	Conf—Neut		0.03	0.01	9.28	***	[0.15,0.04]
				Conf—Unconf		0.04	0.01	−4.04	***	[0.03,0.05]
				Neut—Unconf		0.01	0.01	−13.36	0.127	[−0.00,-0.02]
	Lexical Tone × Speaker Confidence	12.10	0.003	Contour tone	Conf—Neut	0.03	0.01	3.68	***	[0.01,0.05]
					Conf—Unconf	0.02	0.01	2.98	0.009	[0.00,0.04]
					Neut—Unconf	−0.01	0.01	−0.684	1.00	[−0.02,0.01]
				Flat tone	Conf—Neut	0.03	0.01	3.60	0.010	[0.01,0.05]
					Conf—Unconf	0.06	0.01	7.14	***	[0.04,0.07]
					Neut—Unconf	0.03	0.01	3.56	0.001	[0.01,0.05]
Intensity range	Lexical Tone	1.79	0.181	Contour—Flat						
	Speaker Confidence	10.79	0.005	Conf—Neut		0.01	0.00	3.25	0.004	[0.00,0.03]
				Conf—Unconf		0.01	0.00	2.16	0.094	[−0.00,0.02]
				Neut—Unconf		−0.00	0.00	−1.09	0.830	[−0.02,0.01]
	Lexical Tone × Speaker Confidence	7.95	0.020	Contour tone	Conf—Neut	0.03	0.01	3.95	***	[0.01,0.04]
					Conf—Unconf	0.02	0.01	3.38	0.002	[0.01,0.04]
					Neut—Unconf	−0.00	0.01	−0.56	1.00	[−0.02,0.01]
				Flat tone	Conf—Neut	0.00	0.01	0.66	1.00	[−0.01,0.02]
					Conf—Unconf	−0.00	0.01	−0.32	1.00	[−0.01,0.01]
					Neut—Unconf	−0.01	0.01	−0.98	0.988	[−0.02,0.01]
Duration	Lexical Tone	29.29	***	Contour—Flat		0.36	0.07	5.53	***	[0.23,0.49]
	Speaker Confidence	34.55	***	Conf—Neut		0.45	0.08	5.60	***	[0.26,0.64]
				Conf—Unconf		0.07	0.08	0.82	1.00	[−0.13,0.26]
				Neut—Unconf		−0.38	0.08	−4.74	***	[−0.58,-0.19]
	Lexical Tone × Speaker Confidence	0.80	0.796							

^a^Significance levels under Bonferroni-corrections: ^*^p < 0.05; ^**^p < 0.01; and ^***^p < 0.001. ^b^SE, standard error.

The f0 range model revealed a significant effect of Speaker Confidence (see Figure 3B), suggesting that the f0 range was significantly smaller in the neutral-intending expression than the confident and the unconfident expression and the confident did not differ from unconfident expression. The model also revealed a significant effect of Lexical Tone, suggesting that the f0 range was larger in vowels of a contour tone than those of a flat tone. The f0 range model revealed a significant Speaker Confidence x Lexical Tone interaction (see Figure 4B). For vowels of a contour tone, the f0 range was smaller in the neutral-intending than in both the confident and the unconfident expression and the confident did not differ from unconfident expression; for those of a flat tone, the f0 range did not differ among three levels of speaker confidence.

The mean intensity model revealed a significant effect of Speaker Confidence (see Figure 3C), suggesting that the mean intensity was significantly larger in the confident than the unconfident and the neutral-intending expression. No significant difference was shown between the neutral-intending and the unconfident voice. The mean intensity model revealed a significant effect of Lexical Tone, with the mean intensity of vowels of a flat tone sounding more intense than those of a contour tone. Moreover, the mean intensity model showed a significant Speaker Confidence x Lexical Tone interaction (see Figure 4C). For vowels of a contour tone, the mean intensity was larger in the confident than the unconfident and neutral-intending expression. No significant difference was shown between the neutral-intending and the unconfident voice. But for those of a flat tone, the mean intensity differed among all three levels of speaker confidence, with the mean intensity largest in the confident expression, followed by the neutral-intending expression, and lowest by the unconfident.

The intensity range model revealed a significant effect of Speaker Confidence (see Figure 3D), suggesting that the intensity range was significantly larger in the confident than the neutral-intending expression. The main effect of Lexical Tone was not significant. The mean intensity model also showed a significant Speaker Confidence × Lexical Tone interaction (see Figure 4D). For vowels of a contour tone, the intensity range was larger in the confident than the unconfident and the neutral-intending expression, with no difference between the latter two. For those of a lexical tone, the intensity range did not differ among all three levels of speaker confidence.

The duration model was performed on all vowels, with Speaker Confidence and Lexical Tone as two fixed factors, Vowel Item and Speaker as random intercepts. Vowel type (monophthong vs. diphthong) was included as the fixed covariate given that the durations of monophthongs and diphthongs were different. The model revealed a significant effect of Speaker Confidence (see Figure 3E), suggesting that the duration was significantly shorter in the neutrally-intending expression than the confident and the unconfident expression and no significant difference were shown between the latter two conditions. The model revealed a significant main effect of Lexical Tone, suggesting that the normalized duration was significantly larger in vowels of a contour tone than that of a flat tone. The interaction between Speaker Confident and Lexical Tone was not significant.

To conclude, compared with the neutral-intending expression, the speakers raised mean f0, had a greater variation of f0 and prolonged pronunciation time in the unconfident level, while they raised mean intensity, had a greater variation of intensity and prolonged pronunciation time in the confident level. Additionally, considering the interplay of lexical tone and intended confidence, the speaker modulated the mean f0 and mean intensity to a larger extent on the flat tone than the contour tone to differentiate between levels of confidence in the voice, while they modulated the range of f0 and intensity more on the contour tone than the flat tone.

## Discussion

In this study, acoustic-phonetic features at both segmental and suprasegmental level were examined on vowels produced by native Wuxi dialect speakers in confident, unconfident and neutral tone of voice. We found that the intended speaker confidence can be encoded in the mean values of both the first and the second formant at the segmental level. In particular, the vowel spoken in a confident tone demonstrated a larger F1 than the one spoken in neutral and unconfident tones and a larger F2 than the one spoken in a neutral tone. For all vowels, both temporal and spectral prosodic features varied as a function of the intended speaker confidence. Both f0 and intensity measures were associated with the intended speaker confidence. In particular, the more confident the speakers’ intended, the mean f0 was lower and the mean intensity was stronger. As long as the speaker encoded a certain level of confidence, whether confident or not, compared to a neutral tone, the f0 variation was larger and the intensity variation was lower. The speaker modulated the mean f0 and mean intensity to a larger extent on the flat tone than the contour tone to differentiate levels of confidence in voice but, while they modulated the range of f0 and intensity more on the contour tone than the flat tone.

This finding suggests that segmental and suprasegmental features in vowels can provide sufficient information to differentiate when the speakers’ intended high vs. low confidence and when the speaker did or did not intend any emotion or confidence in the sound (Jiang and Pell, 2015). In addition, lexical tone modulated the acoustic encoding of speaker confidence levels in vowels. The speaker modulated mean f0 and mean intensity to a larger extent on the flat tone than the contour tone to differentiate between levels of confidence in the voice but modulated f0 range and intensity range more on the contour tone than the flat tone, suggesting a complex mechanism regarding how tone and vocal expression interplay with each other.

### Encoding speaker confidence in formant features

While previous studies have mostly assigned critical roles of formant peaks in determining vowel identity (Barreda and Nearey, 2011), the current study extended this finding by demonstrating that the formant values can be associated with vocally-expressed confidence in speech production. In particular, speaking in a confident voice raised both F1 and F2.

Existing speech-articulatory models (Fant, 1960; Ladefoged et al., 1978) and empirical studies focusing on the relationship between formant frequencies and tongue positions (Lee et al., 2016) have indicated that the first formant frequency (F1) was typically shown to reflect tongue height, and the F2 was related to the size of the frontal oral cavity or the degree of tongue advancement. The F1 was typically reduced when a high vowel such as /i/ or /u/ pulled the tongue out of pharynx, moved the tongue upward and subsequently increased the volume of the pharynx. The F2 frequency was reduced when the vowel like /a/ or /u/ was produced with the tongue moving far back in the oral cavity (Lieberman and Blumstein, 1988). However, such different articulatory mechanisms underlying F1 and F2 were blurred in a recent study comparing vowels under different consonant contexts (i.e., \h\ + Vowel+\d\ and \d\ + Vowel+\d\ in female speech), which did not demonstrate a universal correlation pattern between tongue positions and formant frequencies. It is shown that F2 is a much more complex reflection of tongue variation in both tongue height and tongue advancement while the F1 variation unambiguously reflects tongue height (Lee et al., 2016).

The relation between formant frequencies and speech articulatory mechanisms allows the possibility for the speaker to encode social-pragmatic meaning, in particular, different levels of confidence in the present study by modulating the articulatory structure and further by moving their tongue positions. Previous works has shown an association between formant placement and speaker emotion. The first and second formants in certain vowels /i/, /u/ and /a/ of 12 emotions varied as a function of the emotional dimension in the tone of voice. While the higher-arousal emotional states resulted in a higher mean values in F1 in all vowels, the positive valence resulted in higher mean values in F2 (Laukka et al., 2005; Goudbeek et al., 2009). The formant encoding of speaker emotion could reflect the articulatory to acoustic mapping. It is likely that the increased feeling of knowing in the confident voice (Guyer J. J. 2016; Jiang and Pell, 2017) could possibly elicit an increased arousal of the speaker, therefore modulating their efforts to articulate vowels by raising the F1 and F2. The formant-frequency values are effectively determined by vowel type (the inter-vowel variability) and vocal tract length (the intra-vowel variability; Turner et al., 2009). Human speakers lower formants by increasing apparent vocal tract length (VTL). They also use formant information to change their own perceived social attributes (e.g., body size, Pisanski et al., 2016) or to perceive the social attributes of others (e.g., speaker height, Barreda, 2016). Accordingly, the innovative finding of this study is that the speaker’s level of confidence influences the change in formants, possibly due to their efforts to encode socio-pragmatic meanings. However, it has also been observed that changes in tongue/lip positions can affect vocal tract length changes. The position of three articulatory parameters appears to contribute significantly to the instantaneous length of the vocal tract: lip, tongue dorsum, and larynx height (Dusan, 2007). The question of whether the resonance peaks encoding the speaker confidence are modulated by the change in VTL or tongue/lip position awaits further explorations with physiological measurements (e.g., MRI). Therefore, although formant cues usually serve as a stable acoustic indicator for distinguishing vowel identity, speakers can encode vocal expression of confidence through these stable characteristics. It is noted that the effect size of the formant characteristics was smaller than that of the prosodic features in the present study, suggesting a relative contribution of segmental vs. suprasegmental features in encoding vocal dynamic cues of speaker confidence (Zhang et al., 2021).

### Encoding speaker confidence in prosodic features

Previous studies have demonstrated the effects of confident voice expressions on suprasegmental features in English spoken sentences (Jiang and Pell, 2014, 2017). The neutrally-intending and confident-intending expression seemed to be differentiated in prosodic cues of vowels, however, the neutrally-intending expression was judged close to confident (Jiang and Pell, 2014) or comparable to confident expression in the believability judgment (Jiang and Pell, 2018). Even though, the perceptual consequences between confident and neutrally-intending voices can be perceptually more similar than between confident and unconfident ones, prosodic marking can be quite distinctive in confident and neutral-intending ones to achieve the speaker’s high feeling of knowing (Jiang and Pell, 2017).

In a dialect with rich tonal possibilities, the suprasegmental pitch encoding of confidence in vowels showed similar mechanisms from that in the longer spoken units. The pattern of mean pitch in vowels of our current results as a function of the intended speaker’s confidence resembled the same patterns in previous studies on sentences based on the perceived level of confidence, with both showing the highest normalized mean f0 in the unconfident level, followed by gradually decreased f0 over the confident and the neutral level. A similar pattern of f0 range also occurred in vowels. Speakers varied f0 to a larger extent when encoding confidence-related information in the voice. These findings suggested that speakers and listeners showed consistency regarding how fundamental frequency encodes speaker feeling of knowing no matter how long the stimuli are.

Past studies have revealed a strong relationship between a speaker’s f0 variation and the perceived attractiveness (Xu et al., 2013), trustworthiness (McAleer et al., 2014), sarcasm (Jansen and Chen, 2020), and speakers’ intended stress (Eriksson et al., 2013) at the lexical or the sentence level inferred from their voice. A further study found that a single word *hello* was enough for the listeners to distinguish speakers of different trustworthiness. The *hello* judged as trustworthy was characterized by a high starting f0 then a marked decrease at mid-utterance to finish on a strong rise (Belin et al., 2017). Additionally, a study asked listeners to judge spoken words of which the pitch contour was manipulated (Ponsot et al., 2018). They showed that sounds rated as trustworthy showed a rapid pitch increase on the second syllable of the word while sounds rated as dominant showed a gradual pitch decrease on both syllables. The modulation of f0 on speakers’ intended confidence was consistent with a view that vocal tract length could serve as a functional role in one’s socio-communicative ability. Speakers can volitionally modulate vocal parameters to imitate voices of different pitches and preferred to adjust f0 (and vocal fold tension in the vocal tract) downward and upward to imitate lower or higher pitched voices when asked to exaggerate body size during speech (Waters et al., 2021). It is suggested that, to encode socio-pragmatic information such as lack of confidence and credibility at the word level, the speaker could mark their voice with more dynamic pitch (Belin et al., 2017; Goupil et al., 2021b).

Our findings on mean intensity and intensity range were generally consistent with the findings on sentence. On vowels in the current study, the normalized mean intensity was higher when the speaker’s intended confidence than lack of confidence or no emotion or confidence. The intensity range was larger when the speaker’s intended high confidence than no emotion or confidence was encoded in the voice. Consistent to the previous studies based on the listener’s perceived, speakers perceived to be unreliable (i.e., uncertain or dishonest) pronounced words with more variable pitch and speech rate, as well as a reduced intensity at the beginning of the word (Goupil and Aucouturier, 2021a; Goupil et al., 2021b). This means that a less certain speaker typically sounded less louder, which could serve as a possible explanation why the intensity of unconfident expressions was smaller than confident expressions. Compared with the neutral expression, speakers varied their voice intensity to a greater extent under either level of confidence (Jiang and Pell, 2017). Like speaker unreliability which was marked by vocal cues of unstable intensity to encode one’s dishonesty and uncertainty, intensity variation can be dramatic to encode speaker levels of confidence.

The pattern on duration showed that speakers were able to use temporal cues to mark to the difference between no intended confidence and intended confidence. Speakers prolonged the pronunciation time when they intended to be confident or unconfident compared with they were refrained from emotions and attitudes. Duration has been associated with communicative meanings (e.g., Speaker persuasiveness: Scherer et al., 1973; Jiang et al., 2017; Speech acts: Hellbernd and Sammler, 2016; Speaker emotion, Banse and Scherer, 1996; Sauter et al., 2010). This finding added novel data to the previous studies on the role of temporal cues on encoding speaker’s confidence information in the small unit of vowels.

### Role of lexical tone in vocal expression of confidence

Despite pitch and loudness were both essential to the encoding of socio-pragmatic meanings (Jiang and Pell, 2017; Caballero et al., 2018; Pell and Kotz, 2021), they seemed to act in concert with the lexical tone to form complex interactive patterns when encoding speaker confidence. A previous study (Zhang et al., 2021) on weighting patterns of different acoustic parameters in encoding prominence in four mandarin tones showed that, on the syllable of flat tones, the mean, maximal and minimal pitch contributed more for marking prominent syllables than mean intensity; while on the syllable of contour tones, the mean intensity and intensity variation weighed higher than pitch-related features. Consistent with these findings, the speaker modulated their mean pitch to a greater extent in the flat tone than the contour tone and demonstrated a stronger modulation of intensity variation in the contour tone than the flat tone to distinguish between the confident and the neutral-intending vowels. Taken together, the speaker tended to modulate mean f0 and intensity levels on the flat tone whereas they tended to vary f0 and intensity level on the contour tone when encoding different levels of communicative meaning. An ERP study investigating the online processing of tone and intonation in Mandarin sentences showed that native Mandarin listeners can distinguish between question intonation and statement intonation when the intonation is associated with a final Tone 4, but fail to do so when the intonation is associated with a final Tone2, which indicated that the processing of intonation can be rapidly influenced by different lexical tones (Liu et al., 2016).

Studies on the interaction between boundary tone and affective prosody showed two patterns how lexical tone and intonation added up: the simultaneous addition of lexical tone of the boundary syllables and sentence intonation or the successive addition of the sentence intonation to the end of the lexical tones instead of simultaneously to the last syllables (Chao, 1933). A previous study (Li et al., 2011) with monosyllabic utterances showed that speakers used a successive addition pattern to express the speakers’ emotion, with the falling successive tone to express disgust and angry and the rising successive tone to express happy and surprise.

According to account of successive addition, the expressive tone was added on the lexical tone by prolonging the duration after the lexical tones are completed. The current findings of longer duration when the speaker expressed confident information compared with the neutral expression suggest that the expressive tone seemed to be successively added to the end of the lexical tones to encode of confidence-related suprasegmental features on different lexical tones. The pattern of successive addition tones in the duration had no difference between the flat tone and the contour tone which indicated the same addition pattern that the expressive tone of confidence was added to both the flat tone and contour tone. Interestingly, the current findings of f0 features suggest that the expressive tone seemed to also affect the f0 contour of the lexical tones. Compared with the neutral-intending expression, the speakers raised mean f0 and had a greater variation of f0 in the unconfident level. Based on the above results, the vocal expression could be added on the lexical tones by a successive addition which was similar to the emotional expressions found in previous studies that were added on the lexical tones by the way of successive addition. Pending more investigations, this finding could expand the successive addition tone account by showing how vocal expression of confidence interacted with lexical tone.

### Limitation and future directions

This study focused on the segmental and suprasegmental representation of speakers’ intended confidence using vowels in a Chinese dialect with a rich tonal system. Dual-route approach of speech communication has assumed the speaker encodes meaning in vocal cues at both linguistic and social level (Sumner et al., 2014; Sumner, 2015). Considering the listeners can automatically and rapidly map of co-present cues (tone, dialect) in speech to recognize social attributes of speakers (Sumner et al., 2014), the speakers due to this reason encode the confidence expression in the segmental and suprasegmental level of vowels. Therefore, the interaction between vocal expression and lexical tones observed on pitch cues provides ingredients to further investigations on how the addition patterns supra-segmental and segmental cues affect listener perception of speaker socio-communicative meanings.

Most previous researches focused on how speakers encode communicative meanings based on standard languages used typically in a formal setting (e.g., English, Mandarin, etc.,), but few has extended the findings to variations of languages typically used in a less-formal setting (e.g., dialect, accented-speech, Jiang et al., 2018, 2020). Comparing native English speakers and English second-language (L2) learners in the acoustic encoding of persuasiveness, a study showed that the consonantal durations, particularly those of continuants, were significantly longer relative to the vowels that followed them when native speakers intended persuasiveness, while for second language learners, the duration of consonants did not change between the neutral-intending and persuasive speech (Banzina, 2021). Speakers of different accents displayed different pronunciation strategies of using phonetic cues in characterizing socio-communicative meanings. In a machine learning experiment of listeners’ perception of confidence and doubt in speakers with different accents, while durational feature contributed to a larger extent in the native accent, the mean and range of intensity contributed more in the foreign and regional accent for the speaker to be perceived with different certainties (Jiang and Pell, 2018). The issue regarding how socio-pragmatic information is encoded in informal dialects and non-standard variations of languages awaits further investigations.

Although the materials were validated by independent listeners, the speakers did not provide their own assessment on the vowels in the current study. In further studies, assessing the self-rated confidence expression after elicitation is necessary to confirm the confidence levels based on speaker’s intention to directly compare how listeners and speakers use vocal cues to decode different levels of speaker confidence.

Future researches could enhance the generalizability of the present findings by adding more speakers considering the limited speakers in the present study and taking into consideration different speech acts and attitudes to dialects. Considering the non-spontaneous elicitation of vowels in the laboratory, the logic follow-up is to do a more naturalistic study by using a spontaneous elicitation procedure, for instance, to respond to the conversational partner with certain communicative.

While a possible articulatory mechanism was inferred based on acoustic results of the current study, the acoustic parameters remained indirect clues. Combined with the role of formant cues in differentiating confident from unconfident and neutral-intending speech, the speech-motor mechanism of the larynx and tongue should be validated to explore the internal articulatory mechanism and its vocal movement through physiological measurement.

## Conclusion

Employing an expression elicitation paradigm for different vocal expression in Wuxi dialect vowels, this acoustic-phonetic study explored the segmental and suprasegmental acoustic representation of confident, unconfident and neutral-intending speech in vowels. Compared with the neutral-intending expression, the speakers raised F1, F2, mean intensity and had a greater variation of intensity in the confident level, while they raised mean f0 and had a greater variation of f0 in the confident level. Additionally, only F1 can distinguish between the confident and unconfident expressions. More importantly, we showed that lexical tone modulated the acoustic encoding of speaker confidence levels in vowels. Specifically, the speaker modulated the mean f0 and mean intensity to a larger extent on the flat tone than the contour tone to differentiate levels of confidence in voice, while they modulated the range of f0 and intensity more on the contour tone than the flat tone. Tonal cues in the Wuxi dialect have an indispensable role in encoding different levels of confidence.

## Data availability statement

The raw data supporting the conclusions of this article will be made available by the authors, without undue reservation.

## Ethics statement

The studies involving human participants were reviewed and approved by Ethics Committee in Institute of Lingusitics, Shanghai International Studies University. The patients/participants provided their written informed consent to participate in this study.

## Author contributions

YJ wrote the first manuscript, performed experiments, and analyzed data for this manuscript. YH analyzed the data, edited the manuscript, and prepared the figures. XJ supervised the study and edited the manuscript. All authors contributed to the article and approved the submitted version.

## Funding

This study was sponsored by Natural Science Foundation of China (31971037), the Shanghai Planning Office of Philosophy and Social Sciences (2018BYY019), the “Shuguang Program” supported by Shanghai Education Development Foundation, Shanghai Municipal Education Committee (20SG31), and Major Program of National Social Science Foundation of China (No. 18ZDA293).

## Conflict of interest

The authors declare that the research was conducted in the absence of any commercial or financial relationships that could be construed as a potential conflict of interest.

## Publisher’s note

All claims expressed in this article are solely those of the authors and do not necessarily represent those of their affiliated organizations, or those of the publisher, the editors and the reviewers. Any product that may be evaluated in this article, or claim that may be made by its manufacturer, is not guaranteed or endorsed by the publisher.

## Footnotes

^1^All but three vowels ([ɚ, uã, ia]) corresponds to a real word in flat tone (see Supplementary Table 1 in Supplementary material). During production, speakers were shown words for both flat and contour conditions. For the vowels with no word mapping in the flat tone, the speaker was shown words in the counterpart of the contour tone but was instructed to produce that in the flat tone of voice.

^2^ https://github.com/feelins/Praat_Scripts/tree/master/10-get_duration_and_formant

^3^We did not include duration values of diphthongs in statistic models because labeling the transition boundary and the boundaries for the stable portion of the vowel articulation could be arbitrary.

^4^y refers to the dependent factor (the acoustic features, e.g., F1, F2, f0, intensity and duration) in each model.

^5^The sample size per cell for all features except for duration was 120 (=4 speakers * (10 monophthongs +10 diphthongs *2 parts of the vowels)). We divided the diphthong into two portions of the vowel to calculate the acoustic features from each separate vowel.

^6^The sample size per cell for the duration was 80 (=4 speakers * (10 monophthongs +10 diphthongs)).

^7^The parametric bootstrapping approach showed an advantage in dealing with statistic issues with a small-sample design (Fisher and Hall, 1991); Also see the link: https://www.millerwjr.com/all-projects/2018/3/10/non-parametric-bootstrap-in-r-with-correction-for-bias-and-skew#:~:text=Parametric%20bootstrapping%20maintains%20an%20advantage%20over%20non-parametric%20bootstrappinghe%20smoothing%20effects%20offered%20by%20estimating%20the%20distribution.
