# Supplementary material for: Segmental and suprasegmental encoding of speaker confidence in Wuxi dialect vowels
Source: Front Psychol. 2022 Dec 12;13:1028106. doi: 10.3389/fpsyg.2022.1028106 (PMC9791101; doi:10.3389/fpsyg.2022.1028106)
Supplement: Supplementary file 1 [file Table_1.DOCX]

Supplementary Material

# Supplementary Figures and Tables

## Supplementary tables

**Table S1** Word list for Wuxi dialect pronunciation.

| Monophthongs | i | u | y | ɚ | a | ʌ | ʊ | ɛ | ã | ɒ̃ |
| --- | --- | --- | --- | --- | --- | --- | --- | --- | --- | --- |
| Contour Tone | 艺 | 画 | 芋 | 饵 | 矮 | 号 | 汗 | 咸 | 杏 | 项 |
| Flat Tone | 医 | 乌 | 淤 | 饵* | 压 | 凹 | 欢 | 淹 | 樱 | 盎 |

| Diphthongs | ia | | ua | iʌ | yʊ | uɛ | ei | əɯ | iã | uã | uɒ̃ |
| --- | --- | --- | --- | --- | --- | --- | --- | --- | --- | --- | --- |
| Contour Tone | 夜 | | 坏 | 摇 | 圆 | 还 | 候 | 河 | 仰 | 横 | 黄 |
| Flat Tone | | 夜* | 蛙 | 妖 | 冤 | 弯 | 欧 | 齁 | 秧 | 横* | 汪 |

Notes:* Some vowel articulations do not have a corresponding real words in the flat tone. In such case, the speakers were instructed to pronounce the vowel only with the flat tone instead of the contour tone as shown in the character.
